# AMPK protects endothelial cells against HSV-1 replication via inhibition of mTORC1 and ACC1

**DOI:** 10.1128/spectrum.00417-23

**Published:** 2023-09-13

**Authors:** Heena Doshi, Katrin Spengler, Amod Godbole, Yi Sing Gee, Jonathan Baell, Jonathan S. Oakhill, Andreas Henke, Regine Heller

**Affiliations:** 1 Institute for Molecular Cell Biology, Center for Molecular Biomedicine, Jena University Hospital, Jena, Germany; 2 Medicinal Chemistry, Monash Institute of Pharmaceutical Sciences, Monash University, Parkville, Victoria, Australia; 3 School of Pharmaceutical Sciences, Nanjing Tech University, Nanjing, China; 4 Metabolic Signaling Laboratory, St. Vincent’s Institute of Medical Research, Fitzroy, Victoria, Australia; 5 Exercise and Nutrition Research Program, Mary MacKillop Institute for Health Research, Australian Catholic University, Melbourne, Victoria, Australia; 6 Section of Experimental Virology, Institute of Medical Microbiology, Jena University Hospital, Jena, Germany; University of Nevada Reno, Reno, Nevada, USA

**Keywords:** HSV-1, AMPK, MK-8722, ACC, mTORC, endothelial cells, antiviral strategies

## Abstract

Herpes simplex virus type 1 (HSV-1) is a widespread contagious pathogen, mostly causing mild symptoms on the mucosal entry side. However, systemic distribution, in particular upon reactivation of the virus in immunocompromised patients, may trigger an innate immune response and induce damage of organs. In these conditions, HSV-1 may infect vascular endothelial cells, but little is known about the regulation of HSV-1 replication and possible defense mechanisms in these cells. The current study addresses the question of whether the host cell protein AMP-activated protein kinase (AMPK), an important metabolic sensor, can control HSV-1 replication in endothelial cells. We show that downregulation of the catalytic subunits AMPKα1 and/or AMPKα2 increased HSV-1 replication as monitored by TCID_50_ titrations, while a potent AMPK agonist, MK-8722, strongly inhibited it. MK-8722 induced a persistent phosphorylation of the AMPK downstream targets acetyl-CoA carboxylase (ACC) and the rapamycin-sensitive adaptor protein of mTOR (Raptor) and, related to this, impairment of ACC1-mediated lipid synthesis and the mechanistic target of the rapamycin complex-1 (mTORC1) pathway. Since blockade of mTOR by Torin-2 as well as downregulation of ACC1 by siRNA also decreased HSV-1 replication, MK-8722 is likely to exert its anti-viral effect via mTORC1 and ACC1 inhibition. Importantly, MK-8722 was able to reduce virus replication even when added after HSV-1. Together, our data highlight the importance of endothelial cells as host cells for HSV-1 replication upon systemic infection and identify AMPK, a metabolic host cell protein, as a potential target for antiviral strategies against HSV-1 infection and its severe consequences.

**IMPORTANCE:**

Herpes simplex virus type 1 (HSV-1) is a common pathogen that causes blisters or cold sores in humans. It remains latent in infected individuals and can be reactivated multiple times. In adverse conditions, for instance, in immunocompromised patients, HSV-1 can lead to serious complications such as encephalitis, meningitis, or blindness. In these situations, infection of endothelial cells lining the surface of blood vessels may contribute to the manifestation of disease. Here, we describe the role of AMP-activated protein kinase (AMPK), a potent regulator of cellular energy metabolism, in HSV-1 replication in endothelial cells. While downregulation of AMPK potentiates HSV-1 replication, pharmacological AMPK activation inhibits it by limiting the availability of required host cell macromolecules such as proteins or fatty acids. These data highlight the role of metabolic host cell proteins as antiviral targets and reveal activation of endothelial AMPK as a potential strategy to protect from severe consequences of HSV-1 infection.

## INTRODUCTION

Herpes simplex virus type 1 (HSV-1) is a contagious and ubiquitous pathogen that belongs to the *Alphaherpesvirinae* subfamily of DNA viruses (1). According to WHO, an estimated 3.7 billion people (67%) of the world population below the age of 50 were infected with HSV-1 in 2016 making it a very common and endemic pathogen (2). HSV-1 primarily infects epithelial cells of the skin or mucosa, replicates at the entry site and is then transported through the axons of sensory nerves to sensory ganglions where it takes on its latent state. Upon various stresses and disease situations, HSV-1 reactivates and travels to skin or mucosa or to the central nervous system. Most HSV-1 infections are asymptomatic or mild causing blisters and open sores, but HSV-1 may also lead to complications such as encephalitis, meningitis, or blindness in newborns or immunocompromised individuals (1, 3, 4). Severe HSV-1 infection is often associated with systemic distribution of HSV-1 (5). This may trigger an innate immune response (6), contribute to the severity of primary disease, cause organ damage (7 – 9) and increase the mortality of patients (5). Of note, HSV-1 DNA has been detected in up to 50% of intensive care unit patients, in particular in patients with sepsis or septic shock or with COVID-19 (10 – 15). Viremia leads to exposure of vascular endothelial cells to HSV-1. These cells have been shown to be sites of HSV-1 infection and replication, which in turn triggered increased adhesivity, permeability, release of inflammatory cytokines, and procoagulant properties (16 – 20). The described alterations may promote manifestation of diseases, for instance, endothelial dysfunction may lead to the destruction of the blood-brain barrier and contribute to herpes simplex encephalitis (21, 22). Endothelial cells may also be sites of HSV-1 latency (23). In line with this, persistent infection of vascular cells with HSV-1 has been suggested to foster chronic vascular diseases such as atherosclerosis (24, 25).

Current clinical treatments of HSV-1-induced severe diseases comprise antiviral drugs that act against viral polymerases or viral thymidine kinases. However, due to drug resistance caused by various mutations in the viral genome (26 – 28), new antiviral strategies are required. In this context, host cell proteins that support or interfere with HSV-1 replication may become important targets and understanding the related antiviral mechanisms is a prerequisite for pharmacological approaches. One such host cell protein is the 5′ adenosine monophosphate (AMP)-activated protein kinase (AMPK), a heterotrimeric serine-threonine protein kinase. It consists of a catalytic α-subunit, which determines the protein kinase activity, and the regulatory β- and γ-subunits, all existing as several isoforms (29). AMPK acts as a master regulator of cellular energy pathways by stimulating ATP-producing and inhibiting ATP-consuming processes (30). It is activated upon energy deprivation, which leads to an increase of AMP/ATP and ADP/ATP ratios and triggers binding of AMP to the γ-subunit. This causes allosteric activation of AMPK, promotes phosphorylation of threonine 172 (T172) at the activation loop of AMPKα by liver kinase B1 (LKB1), and prevents dephosphorylation of T172, which together lead to full activation of the enzyme. AMPK is known to regulate fatty acid synthesis and oxidation by phosphorylating acetyl-CoA carboxylase isoforms 1 and 2 (ACC1 and ACC2), respectively (31), to serve as a metabolic checkpoint for cell growth by inhibiting mechanistic target of rapamycin complex-1 (mTORC1), and to play an important role in regulating autophagy (32). AMPK appears also to be involved in the regulation of virus production in host cells, although its exact role is not completely understood and likely depends on virus type and host cells studied. AMPK activity has been shown to be inhibited (33, 34) or activated (35 – 37) by infection with distinct viruses and mostly a repressive role of AMPK was concluded from these observations. While inhibition of AMPK may be a precondition for successful virus replication, its activation may represent a cellular response to restrict virus production. Consequently, the use of nutraceutical or pharmacological AMPK activators as antiviral drugs received attention (34, 35, 38, 39).

Although systemic HSV-1 infection is frequently observed, especially in immunocompromised patients, and may lead to endothelial dysfunction and vascular complications, little is known about the regulation, control, and clinical importance of HSV-1 replication in endothelial cells. Moreover, the importance of distinct host cell proteins in this process and their suitability as pharmacological targets remains largely unknown. In this study, we aimed at characterizing the role of AMPK as a potential target for anti-HSV-1 therapy in endothelial cells. Using genetic approaches and a specific pharmacological AMPK agonist, MK-8722, we demonstrate that AMPK restricts HSV-1 replication in these cells. The protective effect could be attributed to AMPK-mediated impairment of two essential pathways for successful HSV-1 replication, the mTORC1 pathway and the ACC1-mediated fatty acid synthesis. Our data suggest that activation of AMPK has a translational potential and may reduce vascular damage during systemic HSV-1 dissemination.

## RESULTS

### HSV-1 replicates in endothelial cells

**Fig 1:**
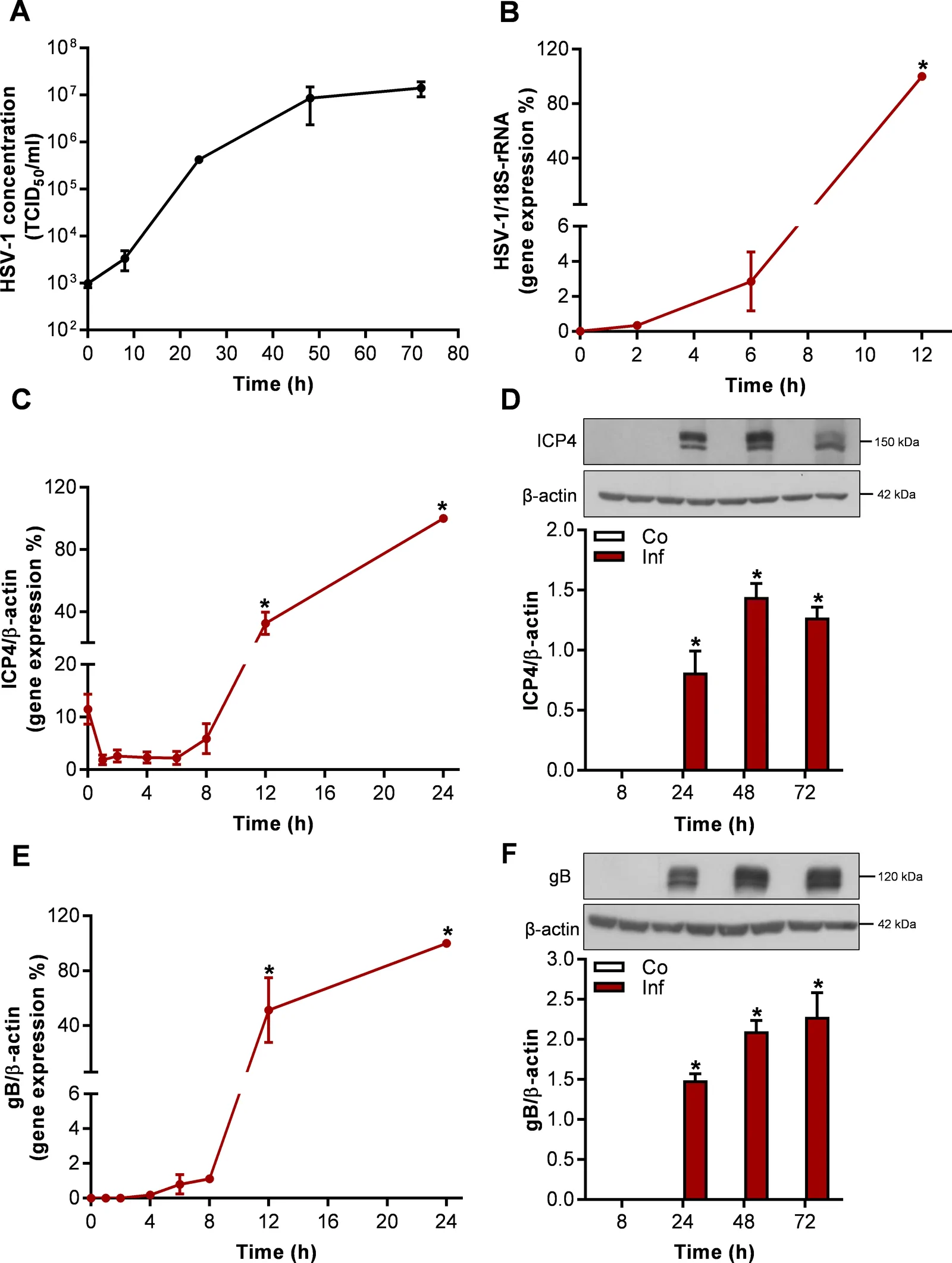
HSV-1 replicates in endothelial cells. (**A and B**) HUVEC were infected with HSV-1 at the dose of 5 multiplicity of infection (m.o.i.) for the indicated times. (**A**) Combined intra- and extracellular virus titers as determined by TCID_50_ assay are shown, *n* = 3. (**B**) DNA was extracted and the amount of HSV-1 DNA relative to 18S-rRNA was determined via qRT-PCR and normalized to cells 12 h p.i., *n* = 5. (**C and E**) HUVEC were infected with HSV-1. After the indicated times RNA was extracted, transcribed into cDNA, and qRT-PCR was performed. Gene expression of ICP4 [infectious cell protein-4 (**C**)] and gB [glycoprotein B (**E**)] were determined via qRT-PCR and normalized to β-actin, *n* = 5. (**C and E**) HUVEC were infected with HSV-1 (Inf) for 8–72 h and lysed. The expression of ICP4 (**D**) and gB (**F**) in western blots was compared to non-infected cells (Co). Representative immunoblots (upper panels) and densitometry analyses normalized to β-actin (lower panels) are shown, *n* = 4. *P < 0.05 vs 0 h using the one-way repeated measurement ANOVA corrected via Holm–Šidák method (**B and D**) or *P* < 0.05 vs non-infected cells using the two-way repeated measurement ANOVA corrected via Holm–Šidák method.

We first characterized HSV-1 replication in endothelial cells. After infection of human umbilical vein endothelial cells (HUVEC) with the HSV-1 KOS strain, samples containing intra- and extracellular virus were obtained at different times post infection (p.i.) and analyzed using tissue culture infectious dose assay (TCID_50_). Fig. 1A shows a time-dependent increase of infectious virus particles up to 72 h p.i. thus demonstrating successful infection and replication of HSV-1 in endothelial cells. We also observed an enhancement of HSV-1 DNA in the nuclear fraction of infected endothelial cells pointing to viral DNA replication (Fig. 1B). Additionally, expression of viral genes and proteins was monitored by real-time PCR and western blotting, respectively, focusing on infectious cell protein-4 (ICP4), an immediate early gene transcription factor expressed by HSV-1 in host cells (40), and glycoprotein B (gB), a late gene transcript required for attachment and entry of HSV-1 into cells (41, 42). ICP4 mRNA was detectable very early after infection and returned to low levels at 1 h p.i. while protein expression was not visible (Fig. 1C; Fig. S1A). After longer periods of infection, likely after one and more virus cycles, both ICP4 mRNA (from 12 h on) and protein (from 24 h on) expression were significantly enhanced (Fig. 1C and D). The mRNA of gB was seen from 4 h p.i. on and showed a time-dependent increase up to 24 h p.i. while gB protein expression was visible between 24 h and 72 h p.i. (Fig. 1E and F; Fig. S1B).

### HSV-1 infection does not change AMPK signaling in endothelial cells

**Fig 2:**
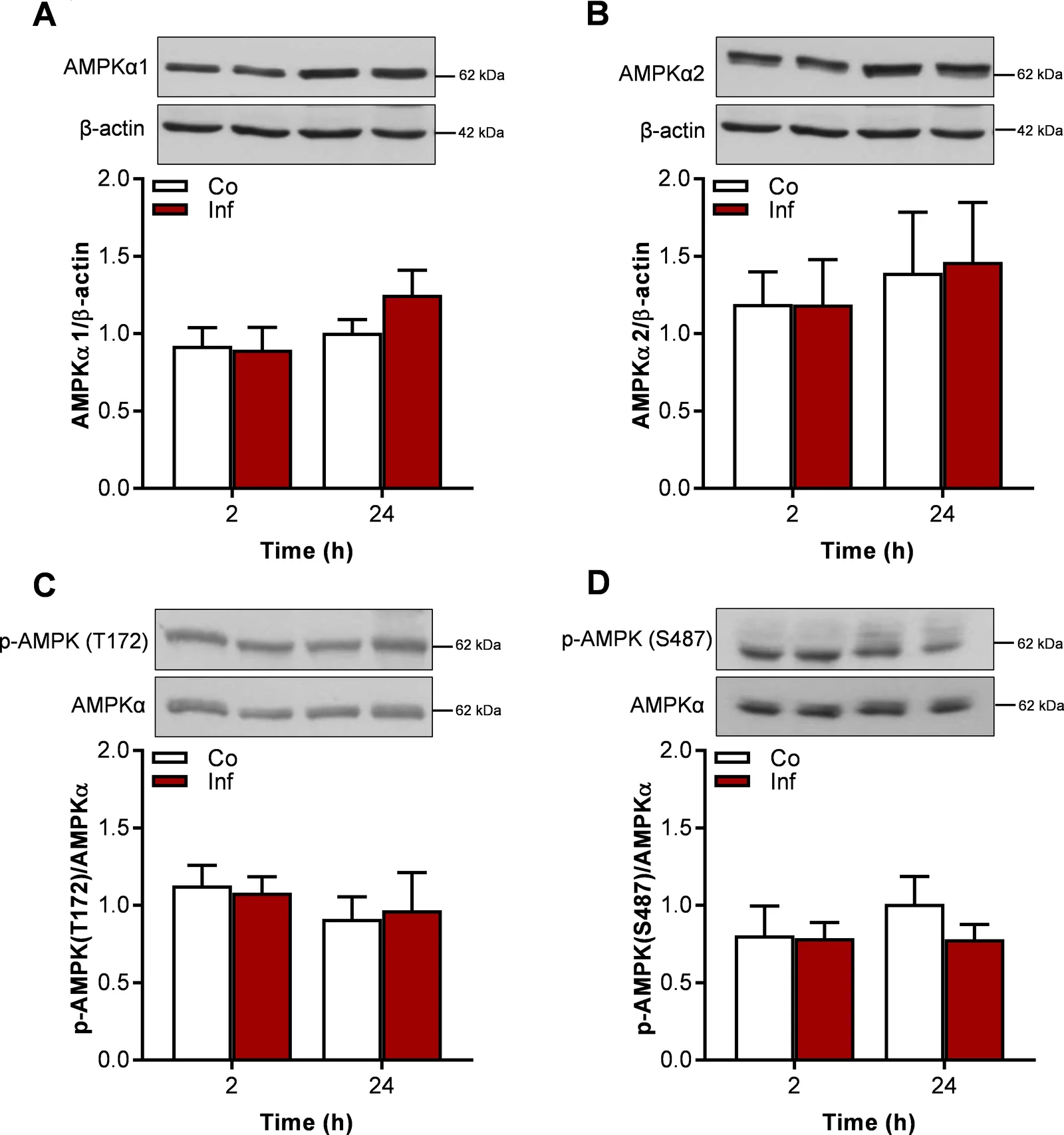
HSV-1 infection does not change AMPK signaling in endothelial cells. HUVEC were infected with HSV-1 (Inf) for 2 or 24 h, lysed, and compared to non-infected cells (Co) in western blot analyses. Representative immunoblots for the expression of AMPKα1 (**A**), AMPKα2 (**B**), and β-actin (all probed from the same blot with intermediate stripping) as well as AMPK phosphorylation at T172 (**C**) or at S487 (**D**) are shown (upper panels). The graphs (lower panels) show densitometry analyses of data normalized to β-actin (**A and B**) or to total AMPKα (**C and D**), respectively, *n* = 3 (**A and B**), *n* = 4 (**C**), *n* = 5 (**D**). No significant alterations were observed using the two-way repeated measurement ANOVA corrected via Holm–Šidák method.

To understand a possible link between AMPK signaling pathways and HSV-1 replication, we examined expression and phosphorylation of AMPK in endothelial cells at 2 h or 24 h p.i. We found that the catalytic subunit isoforms AMPKα1 and AMPKα2 were comparably expressed in infected and non-infected cells (Fig. 2A and B) and did not detect alterations of AMPK phosphorylation at T172 (activation site) or at S487 (inhibitory site) upon HSV-1 infection (Fig. 2C and D). These data indicate that HSV-1 infection and/or replication did not modify AMPK activity in endothelial cells.

### AMPK downregulation promotes HSV-1 replication in endothelial cells

**Fig 3:**
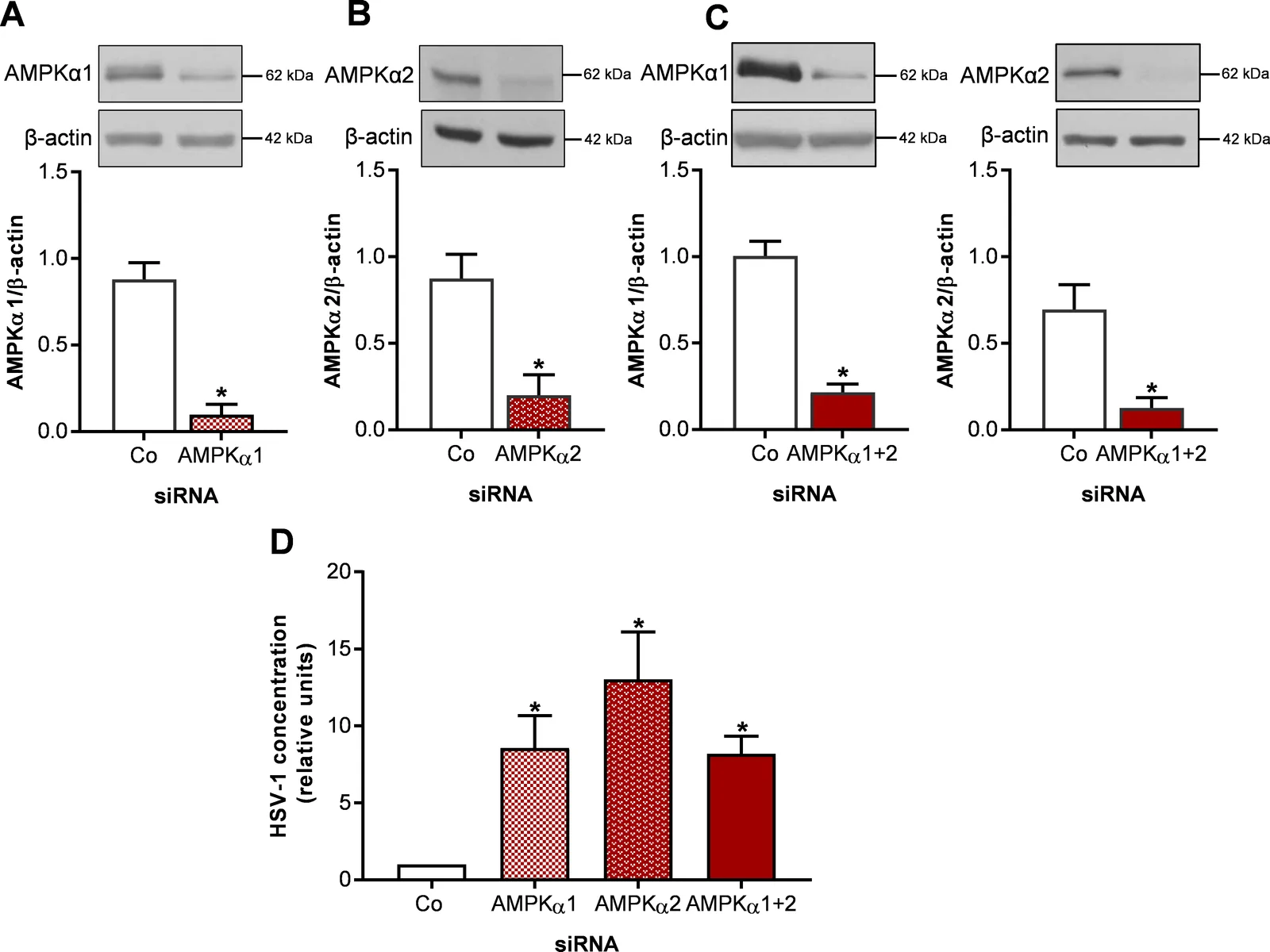
AMPK downregulation promotes HSV-1 replication in endothelial cells. HUVEC were transfected with control, AMPKα1, AMPKα2, or AMPKα1 + 2 siRNAs (0.5 µg/mL, 72 h). Transfected cells were lysed (**A through C**) or infected with HSV-1 for 24 h (**D**). (**A through C**) Efficient downregulation of AMPKα1 and AMPKα2 was proved in western blots. Representative blots and densitometric evaluation are shown, *n* = 5 (**A and B**), *n* = 4 (**C**). (**D**) HSV-1 concentrations were determined by the TCID_50_ assay and normalized to values of control siRNA-treated cells (Co), *n* = 5. *P < 0.05 vs control siRNA-treated cells using the Student‘s *t*-test (**A through C**) or one-way repeated measurement ANOVA corrected via Holm–Šidák method.

Since HSV-1 infection did not change AMPK signaling, we asked whether, *vice versa*, depletion or activation of AMPK affects HSV-1 replication in endothelial cells. We first applied specific siRNAs against the catalytic subunits AMPKα1 or AMPKα2 alone or in combination. With individual targeting, we achieved a downregulation of 88.8% and 77.1% for AMPKα1 and AMPKα2, respectively (Fig. 3A and B). When both siRNAs were given in combination, the downregulation was 77.3% for AMPKα1 and 84.4% for AMPKα2 (Fig. 3C). After infection of these cells with HSV-1 for 24 h, the amount of infectious virus particles was enhanced in AMPK-depleted cells compared to control-siRNA-treated cells regardless of which AMPK isoform was downregulated (Fig. 3D). Thus, the absence of AMPKα potentiates HSV-1 replication suggesting that AMPK may protect endothelial cells from HSV-1. Interestingly, combined downregulation of AMPKα1 and α2 had no additive effect suggesting that both isoforms may target the same downstream pathways but at different levels.

### AMPK activation by MK-8722 restricts HSV-1 replication in endothelial cells

**Fig 4:**
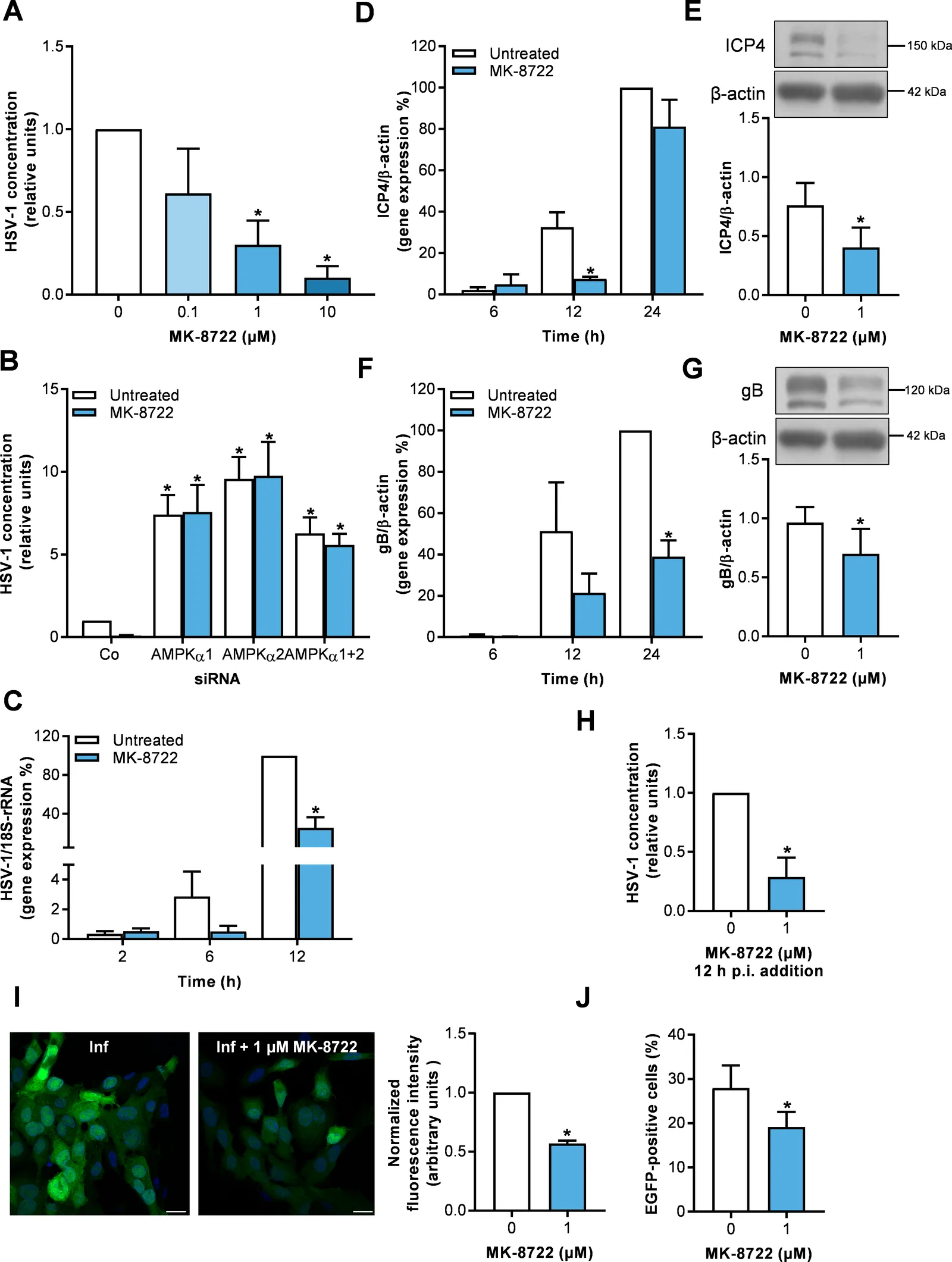
AMPK activation by MK-8722 restricts HSV-1 replication in endothelial cells. (**A**) HUVEC were pretreated with MK-8722 at the indicated concentrations for 1 h and infected with HSV-1 for 24 h. HSV-1 concentration was determined by the TCID_50_ assay and values were normalized to values from non-treated cells, *n* = 5. (**B**) HUVEC were transfected with control-siRNA (Co) or siRNA against AMPKα1, AMPKα2, or AMPKα1 + 2 (0.5 μg/mL, 72 h). Thereafter, cells were incubated in the presence or absence of 1 µM MK-8722 for 1 h and infected with HSV-1 for 24 h. HSV-1 concentrations were determined by the TCID_50_ assay and normalized to values of control-siRNA-treated cells without MK-8722, *n* = 4. (C through G) HUVEC were pretreated with 1 µM MK-8722 for 1 h and infected with HSV-1 for the indicated times (**C, D and F**) or for 24 h (**E and G**). (**C**) DNA was extracted and the amount of HSV-1 DNA relative to 18S-rRNA was determined via qRT-PCR and normalized to untreated cells 12 h p.i., *n* = 5. (**D and F**) RNA was extracted, transcribed into cDNA, and qRT-PCR was performed. Gene expression of ICP4 [infectious cell protein-4 (**D**)] or gB [glycoprotein B (**F**)] were determined via qRT-PCR and normalized to β-actin, *n* = 5. (**E and G**) Cell lysates were analyzed in western blots. Representative immunoblots (deriving from the same batch with intermediate stripping of blots) and densitometry analysis for viral proteins ICP4 (**E**) and gB (**G**) normalized to β-actin are shown, *n* = 5. (**H**) HUVEC were infected with HSV-1 for 12 h, 1 µM MK-8722 was then added to infected cells and virus replication was determined after additional 12 h of incubation. HSV-1 concentration in MK-8722-treated cells as determined by the TCID_50_ assay was normalized to values in non-treated cells, *n* = 5. (**I and J**) HUVEC were pretreated with 1 µM MK-8722 for 1 h and infected with the HSV-1/E70K EGFP strain (10 m.o.i.) for 24 h. (**I**) Representative images show infected HUVEC (Inf) in the absence or presence of 1 µM MK-8722 (left panels). Fluorescence intensity normalized to non-treated infected cells is shown. Hoechst 33342: blue, HSV-1/E70K EGFP: green, scale bar: 20 µM, *n* = 3. (**J**) The number of EGFP-positive cells was determined in flow cytometry, *n* = 5. *P < 0.05 vs non-treated infected cells using one-way repeated measurement ANOVA (**A**) or two-way repeated measurement ANOVA (**C, D and F**) corrected via Holm–Šidák method or using Student‘s *t*-test (E, G, H through J), *P < 0.05 vs respective Co-siRNA-treated cells using two-way repeated measurement ANOVA corrected via Holm–Šidák method (**B**).

We next employed the compound MK-8722, a specific pan-AMPK activator (43) to study the effect of AMPK activation on HSV-1 replication in endothelial cells. We applied conditions (0.1–10 µM, 24 h incubation), which did not affect endothelial cell viability regardless of whether the compound was added to control or infected cells (Fig. S2A). After pretreatment of endothelial cells with 0.1–10 µM MK-8722 for 1 h, cells were infected with HSV-1 for 24 h and the TCID_50_ assay was applied to analyze virus concentrations. As shown in Fig. 4A, MK-8722 led to a strong dose-dependent reduction of virus particles. This effect was largely prevented in cells, in which AMPKα1 and/or AMPKα2 were downregulated confirming that it was mainly mediated by AMPK (Fig. 4B).

To further substantiate the antiviral effect of MK-8722 in endothelial cells, we isolated viral DNA from the nuclear fraction and found a clear reduction by 1 µM MK-8722 at 6 h and 12 h p.i. suggesting an inhibition of nuclear entry and/or HSV-1 DNA replication (Fig. 4C). We also analyzed the impact of MK-8722 on mRNA and protein levels of ICP4 and gB especially at times where significant expression of both had already been observed, which was most probably due to increased virus production (Fig. 1C through F). Pretreatment of cells with 1 µM MK-8722 before infection led to a significant reduction of mRNA and protein expression of both, gB and ICP4, at 12 h as well as at 24 h p.i. as compared to non-treated infected cells (Fig. 4D through G). We tested whether MK-8722 interferes with virus replication when administered after HSV-1 infection to mimic a pathophysiologically relevant situation. Endothelial cells were exposed to HSV-1 for 12 h to allow virus entry, expression of viral genes and DNA replication, then 1 µM MK-8722 was added for further 12 h and samples were processed for the TCID_50_ assay. Fig. 4H shows that MK-8722 led to a significant decrease of infectious virus particles under these conditions suggesting an effect on virus production.

The antiviral effect on HSV-1 replication was confirmed in experiments employing HSV-1/E70K EGFP, a virus encoding enhanced green fluorescent protein (EGFP), although the observed effects were more moderate compared to the HSV-1 strain KOS. This may be related to the different strains and differences in the applied detection methods. However, cells pretreated with 1 µM MK-8722 for 1 h and thereafter infected with HSV-1/E70K EGFP for 24 h showed a significant decrease in fluorescence intensity of around 40% as compared to non-treated infected cells (Fig. 4I) and a reduction of EGFP-positive cells detected by flow cytometry by 30% (Fig. 4J; Fig. S3).

### Inhibition of the mTORC1 pathway and fatty acid biosynthesis by MK-8722 contributes to inhibition of HSV-1 replication

To further explore mechanisms underlying the antiviral effect of MK-8722, we characterized MK-8722-induced activation of AMPK. The experiments were performed in full growth medium, which was employed in all infection studies. MK-8722 is an allosteric AMPK activator. It binds to the allosteric drug and metabolite (ADaM) site between the α-kinase domain and the β-glycogen-binding domain, stabilizes the enzyme’s activation loop in an active conformation and allows bypassing the need for AMPKα T172 phosphorylation for full enzyme activation (44, 45). Thus, AMPK activation by MK-8722 is not reflected by AMPK phosphorylation at T172 but by phosphorylation of AMPK downstream targets such as ACC and rapamycin-sensitive adaptor protein of mTOR (Raptor).

**Fig 5:**
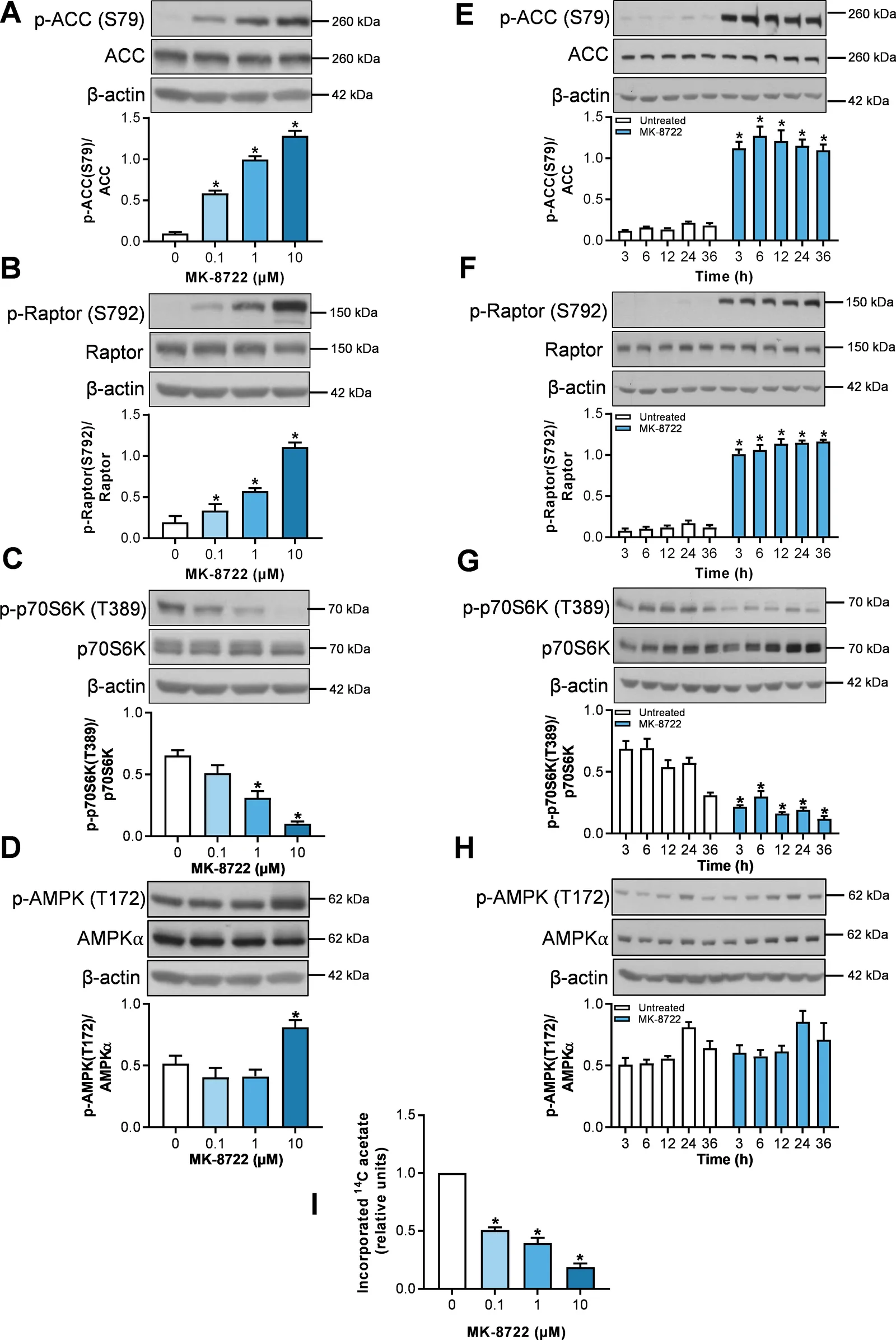
MK-8722 is a strong activator of AMPK. (A through H) HUVEC were treated with the MK-8722 at the indicated concentrations for 24 h (A through D) or with 1 µM MK-8722 for the indicated times (E through H). Cells were lysed and subjected to western blot analyses of ACC phosphorylated at S79 (**A and E**), Raptor phosphorylated at S792 (**B and F**), p70S6K phosphorylated at T389 (**C and G**) and AMPK phosphorylated at T172 (**D and H**), of the respective non-phosphorylated proteins and of β-actin as additional loading control. Representative immunoblots and quantification by densitometry with normalization of phosphorylated proteins to total proteins are presented. Blots shown in A/D, B/C, and E/F derived from the same western blotting experiment with intermediate stripping for staining of target proteins and identical β-actin staining, *n* = 4 (B, E through H), *n* = 5 (**A, C and D**). (**I**) HUVEC were treated with MK-8722 at the indicated concentrations for 24 h. Incorporated [1–14C]-acetate was measured, normalized to the protein content, and is depicted relative to untreated cells, *n* = 4. *P < 0.05 vs non-treated control using one-way repeated measurement ANOVA corrected via Holm–Šidák method (A through D, **I**) or vs time-matched controls using two-way repeated measurement ANOVA corrected via Holm–Šidák method (E through H).

We first looked at the effects of MK-8722 at different concentrations after 24 h of stimulation. Figure 5A and B show that MK-8722 triggered a robust concentration-dependent phosphorylation of ACC and Raptor indicating activation of AMPK. In parallel, the phosphorylation of the mTORC1 substrate p70S6 kinase (p70S6K) was reduced indicating an inhibition of the mTORC1 pathway (Fig. 5C). We then studied the time-dependent activation of the AMPK pathway in response to treatment with 1 µM MK-8722 for 3 h to 36 h. A strong and persistent phosphorylation of ACC and Raptor up to 36 h was seen (Fig. 5E and F) and, in parallel, p70S6K phosphorylation was significantly decreased (Fig. 5G). As expected, AMPK phosphorylation at T172 was only seen with 10 µM MK-8722 (Fig. 5D and H), thus confirming that MK-8722 activation of AMPK does not require phosphorylation of the enzyme (45). In line with the phosphorylation of ACC at its inhibitory site S79, MK-8722 treatment resulted in a significant inhibition of lipid synthesis in endothelial cells. This was shown as a concentration-dependent decrease of labeled acetate incorporation into the fraction of neutral lipids as compared to non-treated cells (Fig. 5I).

**Fig 6:**
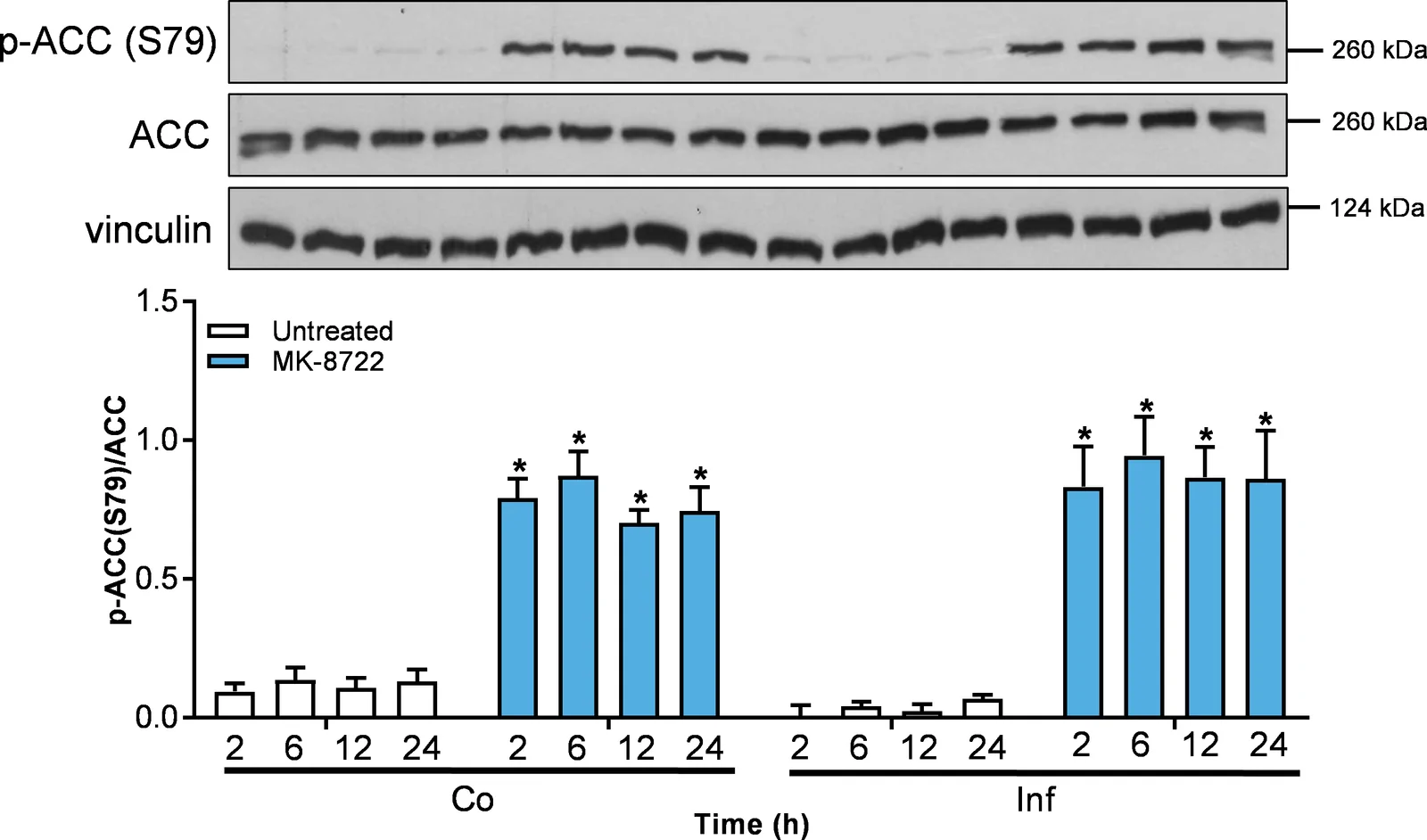
MK-8722-mediated AMPK activation is comparable between non-infected and HSV-1-infected HUVEC. HUVEC were infected with HSV-1 (Inf) or left non-infected (Co) and were subsequently stimulated with 1 µM MK-8722 for the indicated times. Cell lysates were subjected to western blot analyses. Representative immunoblots and densitometry analyses for ACC phosphorylated at S79 normalized to total ACC are shown, *n* = 4. *P < 0.05 vs respective non-treated control using two-way repeated measurement ANOVA corrected via Holm–Šidák method.

**Fig 7:**
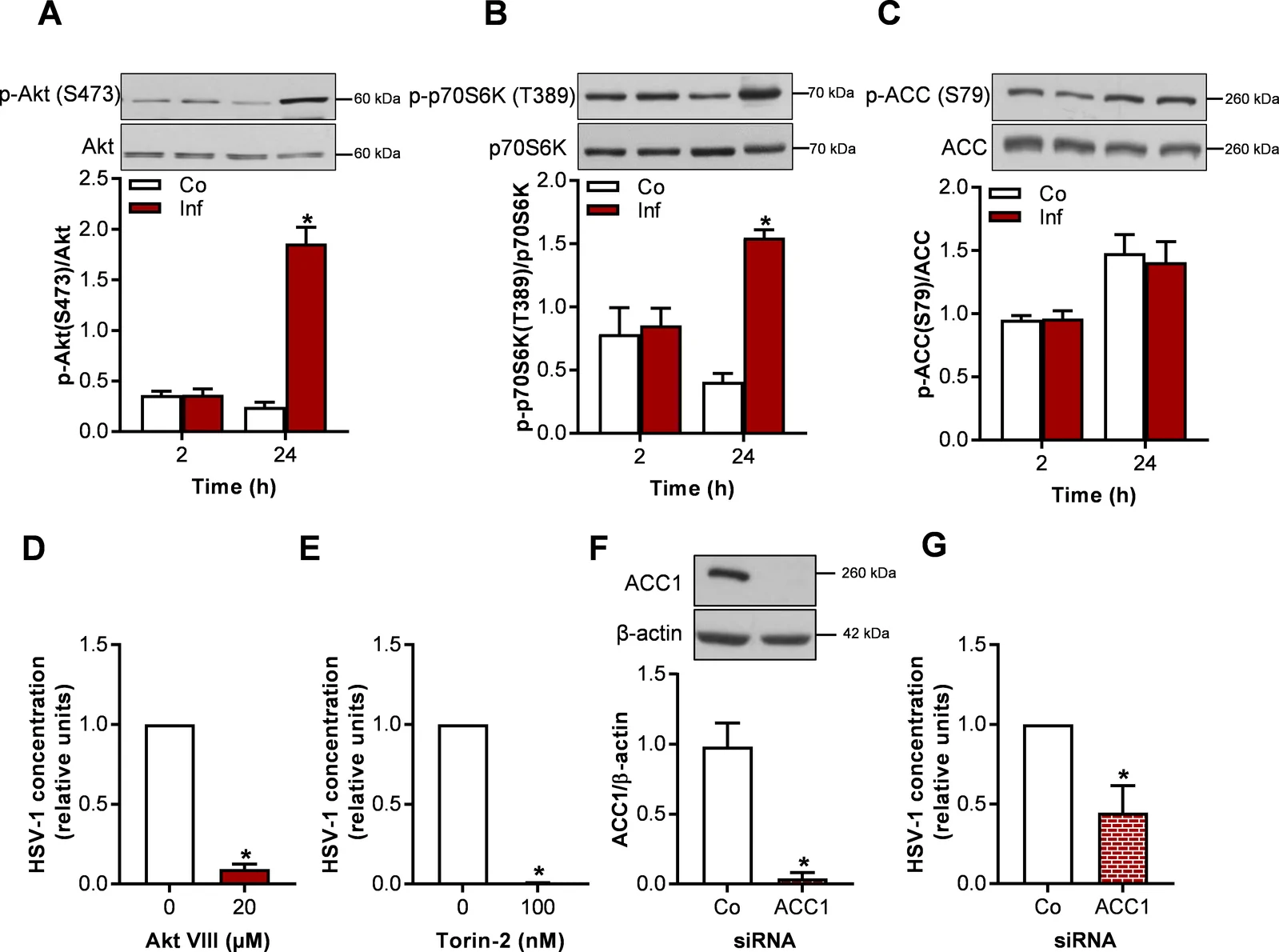
mTORC1 pathway and ACC1 are required for HSV-1 replication in endothelial cells. (A through C) HUVEC infected with HSV-1 (Inf) for 2 or 24 h and non-infected cells (Co) were lysed and subjected to western blot analyses. Representative immunoblots for Akt phosphorylated at S473 (**A**), p70S6K phosphorylated at T389 (**B**) and ACC phosphorylated at S79 (**C**), and densitometry analyses after normalization to the respective total proteins are shown, *n* = 3 (**A and B**), *n* = 6 (**C**). (**D and E**) HUVEC were pretreated for 1 h with 20 µM Akt inhibitor VIII (**D**) or with 100 nM Torin-2 (**E**) prior to HSV-1 infection for 24 h. HSV-1 concentrations were determined by the TCID_50_ assay and normalized to values of untreated infected cells, *n* = 4. (**F and G**) HUVEC were transfected with control- or ACC1-siRNA (0.5 µg/mL, 72 h). Transfected cells were lysed and subjected to western blot analyses (**F**) or infected with HSV-1 for 24 h (**G**). (**F**) Representative blots and densitometry evaluation indicate efficient downregulation of ACC1, *n* = 4. (**G**) Relative HSV-1 concentrations in ACC1-depleted cells normalized to values in control siRNA-treated cells as determined by the TCID_50_ assay are shown, *n* = 5. *P < 0.05 vs non-infected cells (Co) using one-way repeated measurement ANOVA corrected via Holm–Šidák method (**A and B**). *P < 0.05 vs untreated infected controls (**D and E**) or control-siRNA-treated cells (**F and G**) using the Student‘s *t*-test.

To verify that MK-8722 efficiently activates AMPK in infected cells as well, we monitored phosphorylation of ACC, which is not affected by HSV-1 infection itself (shown in Fig. 7C). Figure 6 demonstrates that MK-8722 (1 µM, 2–24 h) is equally potent in stimulating phosphorylation of ACC in control cells and infected cells.

To understand which target may be responsible for the AMPK-mediated protection against HSV-1 replication, we investigated whether HSV-1 infection of endothelial cells affects mTOR or ACC pathways and whether inhibition of mTOR or downregulation of ACC1 modifies virus replication. We found a significantly increased phosphorylation of protein kinase B (PKB/Akt), an upstream kinase of mTOR, and of p70S6K, an mTORC1 target, at 24 h p.i. (Fig. 7A and B) indicating that HSV-1 stimulates the mTORC1 pathway via Akt. In line with the failure of HSV-1 to activate AMPK (Fig. 2C), no changes in ACC phosphorylation were seen (Fig. 7C). We then applied inhibitors to block the mTOR pathway with Akt VIII, an inhibitor of Akt (46), or with Torin-2, a selective ATP-competitive inhibitor of mTOR (47). Both inhibitors were shown not to affect viability of non-infected or infected endothelial cell under the chosen conditions (20 µM Akt VIII or 100 nM Torin-2, preincubation for 1 h, 24 h) (Fig. S2B and C). They led to a significant suppression of HSV-1 replication at 24 h p.i. as compared to non-treated infected cells (Fig. 7D and E). Similarly, downregulation of ACC1 by specific siRNA (95.8% reduction, Fig. 7F) led to strong inhibition of HSV-1 production in comparison to cells treated with control siRNA (Fig. 7G). Thus, both, Akt/mTOR and ACC1 pathways are involved in efficient HSV-1 replication in endothelial cells. Collectively, these data propose that MK-8722 exerts an antiviral effect via AMPK-dependent inhibition of mTORC1 and ACC1-mediated pathways, which are required for virus replication in host cells.

## DISCUSSION

HSV-1 is a highly infectious and ubiquitous pathogen, which along with its localized primary infection may lead to systemic distribution and infection of vascular endothelial cells (17, 48, 49). The latter may contribute to serious complications of HSV-1 infection such as HSV-1 encephalitis, a widespread sporadic acute encephalitis (50, 51). In the present study, we show that pharmacological activation of AMPK, a key regulator of cellular metabolism, restricts HSV-1 replication in endothelial cells by inhibiting fatty acid synthesis and the mTORC1 pathway. Inhibition of virus replication also occurs when AMPK is activated post-infection suggesting a translational potential of our findings.

In line with previous studies (20), our data demonstrate that HSV-1 replicates in endothelial cells as measured by intra- and extracellular virus titers, endothelial expression of the HSV-1 genes ICP4 and gB, and an increase of HSV-1 DNA in the nuclear fraction. The observed kinetics of viral gene expression match previous observations in different cell lines (40, 52, 53), although initial values for the immediate early gene ICP4 transcript were low. ICP4 mRNA shows a small transient increase right after infection of cells and a significant rise from 12 h p.i. on while the expression of gB mRNA started at 6–8 h p.i. and was continuously increasing. At early times after infection, protein expression of ICP4 or gB was not detectable probably due to sensitivity problems based on low transcript levels and detection limits of western blotting. Protein expression was only seen from 24 h p.i. on, which most probably corresponds to an increased amount of newly formed virus particles at this time. In line with this, we observed significant virus titers between 24 and 72 h p.i.

In contrast to previous observations, HSV-1 infection has not altered the AMPK phosphorylation state in endothelial cells in this study. In neurons, a transient activation of AMPK by HSV-1 had been observed (39) and in several cell lines, HSV-1 infection inhibited AMPK via a mechanism involving the viral restriction factor Tudor domain-containing 7 (TDRD7) protein (38, 54). Thus, modulation of AMPK activity by HSV-1 may be cell-specific and possibly related to the expression and importance of TDRD7. Contrasting observations on the modulation of AMPK activity by viral infections were also made with respect to other virus species and cell types. For instance, Zika virus led to an inhibition of AMPK phosphorylation in endothelial cells, thereby allowing a glycolytic response required for virus replication, although this was mainly observed at later infection states (>48 h p.i.) (34). Hepatitis C virus (HCV) inhibited AMPK via an Akt-dependent phosphorylation at S487 promoting lipid accumulation necessary for HCV replication in a hepatocyte cell line (33). On the other hand, AMPK activation and subsequent modulation of lipid metabolism was seen in response to Coxsackievirus B3 in primary myocardial cells and after Hepatitis B virus or Dengue virus infection in liver cell lines where AMPK was seen as a negative feedback regulator (35, 36) or even as a supporter of virus replication (37). Together, these data demonstrate that the response of the AMPK pathway has to be individually characterized in each infection system.

To study the role of AMPK in HSV-1 replication, we employed the RNAi technology to downregulate catalytic AMPKα subunits in endothelial cells. AMPK is known to exist as a heterogeneous pool of isoenzymes of different subunit composition but so far, the role of AMPK isoforms in virus replication has not been studied. In endothelial cells, AMPKα1 is known to be the predominant isoform and to mediate important endothelial functions such as VEGF-induced angiogenesis, autophagy, and glycolysis (55 – 57). AMPKα2 represents the minor isoform, but is still known to contribute to endothelial homeostasis by counteracting different cellular stresses (58 – 61). We found that siRNA-mediated depletion of either catalytic AMPK subunit, that is, AMPKα1 or α2, led to an increase of HSV-1 replication in endothelial cells suggesting that both isoforms are negative regulators of virus production. Interestingly, inhibition of virus replication by combined downregulation of AMPKα1 and α2 subunits did not exceed the effect of individual knockdown. One possible explanation could be that both isoforms affect the same pathways but at different levels, which may be related to their distinct subcellular localization. For instance, AMPKα2, which exhibits a preferential nuclear localization (62) may be involved in regulating gene expression, while AMPKα1, which does not contain a nuclear localization sequence (63), may control the activity of proteins in the cytoplasm.

To substantiate the potential role of AMPK in HSV-1 replication, we applied MK-8722, a pharmacological pan-AMPK activator (43). Treatment of endothelial cells with MK-8722 lowered HSV-1 virus titers, decreased HSV-1 DNA in the nuclear fraction, and led to reduced fluorescence intensity and a lower number of EGFP-positive cells after infection with HSV-1/E70K EGFP, a virus encoding EGFP (64). MK-8722-treated cells also showed a reduced expression of the HSV-1 genes ICP4 and gB and their respective gene products but only at later time points of infection thus rather referring to a decrease of newly formed virus particles. Of note, MK-8722 inhibited HSV-1 replication in endothelial cells also when added after virus infection pointing to a translational potential of our findings. The latter data let us conclude that MK-8722 most probably does not affect virus attachment and entry. An effect on immediate early and late gene expression at times where they are first expressed in the virus cycle could also not be shown, although it cannot be excluded. Based on our data, however, we suggest that MK-8722 via AMPK may impact DNA replication and, maybe, stages thereafter such as virion packaging and spread, which has to be clarified in future studies. Together, MK-8722 revealed to be a potent inhibitor of HSV-1 replication in endothelial cells confirming the suggested protective role of AMPK against HSV-1. In line with this, MK-8722 did not inhibit HSV-1 replication in cells, in which AMPKα1 and/or AMPKα2 were downregulated confirming that the antiviral effect was dependent on AMPK. Interestingly, depletion of either AMPKα subunit was sufficient to rescue the inhibitory effect of MK-8722 again suggesting that AMPKα1 and α2 may target different proteins of the same regulatory pathways.

We could verify that MK-8722 potently activates the AMPK pathway under the experimental conditions of this study even when applied to already infected cells. Since MK-8722 is an allosteric activator, which can bypass the requirement for AMPK phosphorylation at T172, downstream targets of AMPK need to be studied to monitor its potency. We found a strong and persistent phosphorylation of two AMPK downstream targets, ACC (both in control and infected cells) and Raptor (studied in control cells only since HSV-1 affects the mTOR pathway itself), for up to 36 h. Both ACC and Raptor could play a role in HSV-1 replication in endothelial cells. ACC1 regulates fatty acid synthesis, which may be needed to supply building blocks for generating the HSV-1 lipid bilayer envelope (65 – 67) while Raptor controls the mTORC1 pathway, which is suggested to be implicated in viral protein translation and growth (68, 69). AMPK is known to inhibit both, ACC1-mediated fatty acid synthesis (31) and the mTORC1 pathway by phosphorylating ACC1 or the mTORC1 regulators’ tuberous sclerosis complex 2 (TSC2) and Raptor (70). Accordingly, MK-8722 led to a robust inhibition of lipid synthesis in our study monitored as incorporation of radiolabeled acetate into the neutral lipid fraction. In addition, it induced a strong decrease in phosphorylation of p70S6K, a downstream target of mTORC1.

To understand whether the antiviral effect of MK-8722 is related to inhibition of mTORC1 or ACC1, we studied the role of these proteins in HSV-1 replication in endothelial cells. To this end, pharmacological inhibitors of the Akt/mTOR pathway and specific siRNA against ACC1 were employed. Our data show that inhibition of Akt known to trigger mTORC1 activation by Akt inhibitor VIII or inhibition of mTOR by Torin-2 reduced the formation of infectious virus particles in endothelial cells indicating that the Akt/mTORC1 pathway is essential for successful replication. In line with this, the Akt-mTORC1 pathway was upregulated by HSV-1 at later times p.i. as shown by increased phosphorylation of both Akt and p70S6K.

These data agree with previous studies in epithelial cell lines and neurons showing activation of Akt by HSV-1 (71, 72) or reduction of virus entry by Akt inhibition (73). The importance of the mTOR pathway for HSV-1 propagation is also highlighted by the fact that mTORC1 activation is maintained in infected cells even under conditions of amino acid or energy deficiency by employing virus-encoded mTORC1 regulators (74, 75). For instance, the HSV-1 UL46 gene product, which stimulates phosphoinositide 3-kinase, and the Us3 kinase, which masquerades as Akt without underlying Akt regulatory pathways (76), have been shown to support virus reproduction during amino acid withdrawal (75). Furthermore, Us3 has been reported to mediate sustained mTORC1 signaling during energy insufficiency, in which mTORC1 is normally inhibited by AMPK (74). Us3 like Akt phosphorylates TSC2, an mTORC1 suppressor, at its residues S939 and T1462 leading to activation of the Rheb-mTORC1 axis (76). On the other hand, AMPK phosphorylates TSC2 on residues T1227 and S1345 resulting in inactivation of the small GTPase Rheb and subsequently to inhibition of its downstream target mTORC1 (32). In HSV-1-infected fibroblasts, Us3-mediated TSC2 phosphorylation was able to override the effect of AMPK, thereby allowing virus production under conditions of energy deficiency (74). Competition between Us3 and AMPK may be functional in endothelial cells as well and may explain, in part, the increased virus production in AMPK-deficient cells. However, a long-lasting pharmacological AMPK activation of up to 36 h, as shown for MK-8722 may reverse conditions and lead to downregulation of the mTOR pathway as seen in our study. Given the reported importance of Us3 for mTORC1 activation in HSV-1-infected cells, the question arises as to the significance of the endogenous Akt in this process. In our study, we show both, an activating phosphorylation of Akt and inhibition of virus production by Akt inhibitor VIII, a specific allosteric inhibitor of Akt. Since the latter has been shown not to inhibit Us3 (76), our data suggest that Akt-mediated pathways may also play a role in HSV-1 replication. HSV-1 may activate mTORC1 through both Akt-dependent and Us3-dependent mechanisms as suggested previously (76) and not all Akt substrates may be targeted by Us3. Since Akt activation occurred late after HSV-1 infection, it might be required for late stages in the replication cycle, but further studies have to address these questions. On the other hand, it should be noted that Us3 substrates do not only include Akt targets but also PKA targets and Erk (77, 78).

In addition to the mTORC1 pathway, fatty acid synthesis controlled by ACC1 was required for HSV-1 replication in endothelial cells. Early studies had already observed lipid accumulation in response to HSV-1 infection (49). We here show that downregulation of ACC1 using an RNAi approach led to a significant decrease in virus titers, thus revealing the ACC1 pathway as another metabolic pathway involved in HSV-1 replication. Thus, two downstream targets of AMPK, mTORC1 and ACC1, are essential for HSV-1 replication, most probably by affecting virus protein translation and generation of building blocks for virion packaging. Both have been shown to be inhibited by MK-8722 in this study indicating that the antiviral effect of MK-8722 is mediated through inhibition of ACC1 and mTORC1. These results add to previous data showing that the AMPK/sirtuin1 axis is responsible for the inhibition of HSV-1 replication in neurons (39).

In summary, using genetic and pharmacological approaches our study describes the regulatory role of AMPKα1 and α2 isoforms against HSV-1 replication in endothelial cells, which occurs, for instance, in disseminated infections in immunocompromised patients. We specifically show that the pan-AMPK activator MK-8722 added before or after HSV-1 infection is a potent inhibitor of virus replication, which correlates with its strong ability to activate AMPK and to inhibit mTORC1 and ACC1 pathways downstream of AMPK. Our study highlights the importance of anabolic pathways in host cells as potential targets to interfere with virus replication.

## MATERIALS AND METHODS

### Chemicals

M199 and Eagle’s minimum essential medium (EMEM) were purchased from Lonza (Basel, Switzerland). Fetal calf serum (FCS), human serum, endothelial cell supplement (ECGS), trypsin-EDTA, non-essential amino acids, L-glutamine, anhydrous dimethylsulfoxide (DMSO), Akt inhibitor VIII (Akt VIII), Torin-2, and Hoechst 33342 were from Sigma (Taufkirchen, Germany). Bovine serum albumin-C (BSA-C) was obtained from Aurion (Wageningen, The Netherlands) and goat serum from Cell Signaling Technology (Frankfurt, Germany). EDTA-free protease inhibitor cocktail was purchased from Roche Diagnostics (Mannheim, Germany). β-Mercaptoethanol, dithiothreitol (DTT), EDTA, bovine serum albumin, Tween-20, and Triton X-100 were obtained from Carl Roth GmbH (Karlsruhe, Germany). Fluoromount-G was from Southern Biotech (Birmingham, AL, US). MK-8722 was purchased from Aobious (Gloucester, MA, USA). The siRNAs against AMPKα1 or AMPKα2, ACC1, and non-targeting control-siRNA were SMARTpool-siRNAs obtained from Horizon, Dharmacon RNAi, and Gene Expression (Lafayette, CO, USA).

### Antibodies

Rabbit monoclonal antibodies for western blotting against β-actin (#4970), vinculin (#4650), AMPKα (#2532), AMPKα1 (#2795), AMPKα2 (#2757), ACC1 (#3676), p70S6K (#9202), Raptor (#2280), Akt (#9272) and against phospho-AMPK [T172 (#2535) or S485 (#2537)], phospho-ACC (S79) (#3661), phospho-p70S6K (T389) (#9205), phospho-Raptor (S792) (#2083), and phospho-Akt (S473) (#9271) were obtained from Cell Signaling Technology (Frankfurt, Germany). Mouse monoclonal antibodies against the HSV-1 proteins gB (ab6506) and ICP4 (ab6514) were obtained from Abcam (Cambridge, United Kingdom).

### Primers for qPCR

The primers used for qPCR were obtained from Sigma (Taufkirchen, Germany) and the sequences are provided in Table 1.

**TABLE 1:** The primers used for qPCR

Target	Sequence
1. ICP4	Forward: GCGTCGTCGAGGTCGT Reverse: CGCGGAGACGGAGGAG
2. gB	Forward: GGACATCAAGGCGGAGAACA Reverse: TTCTCCTTGAAGACCACCGC
3. HSV-1 UL42 region	Forward: ACGACGACGTCCGACGGCGA Reverse: GTGCTGGTGCTGGACGACAC
4. β-actin	Forward: GGGACGACATGGAGAAAATCTG Reverse: GAAGGTCTCAAACATGATCTGGG
5. 18S-rRNA	Forward: CGAACCTCCGACTTTCGTTCT Reverse: CGCCGCTAGAGGTGAAATTCT

### Cells

Endothelial cells (HUVEC) were isolated from veins of anonymously acquired umbilical cords according to the Declaration of Helsinki “Ethical Principles for Medical Research Involving Human Subjects” (1964) as previously described (57). The Jena University Hospital Ethics Committee approved the protocol. Donors were informed and gave written consent. For cell preparation, umbilical cord veins were washed with 0.9% NaCl solution and then incubated at 37°C for 3 min with 0.01% collagenase dissolved in M199. After rinsing the veins with M199/10% FCS, detached cells were centrifuged at 500 × *g* for 6 min. The pellet was resuspended in M199/10% FCS and cells were seeded on a cell culture flask coated with 0.2% gelatin. After 24 h, cells were washed and further cultured in full growth medium (M199, 17.5% FCS, 2.5% human serum, 7.5 µg/mL ECGS, 7.5 U/mL heparin, 680 µM glutamine, 100 µM vitamin C, 100 U/mL penicillin, 100 g/mL streptomycin) at 37°C and 5% CO_2_. If not otherwise indicated, cells from the second passage were seeded at a density of 27,500/cm^2^ and used for experiments three days after seeding. For transfection with siRNA, the seeding density was 23,000/cm^2^. Immunofluorescence studies were performed with primary endothelial cells seeded on coverslips (50,000 cells/cm^2^). Most experiments were performed with cells on 30 mm culture dishes or 24-well plates.

For viral titration experiments, African Green Monkey Kidney cells (GMK, Vero, ATCC number: CCL-81) were used as reporter cells. These cells are highly susceptible to virus infections as they lack interferon response production. They were cultured at 37°C and 5% CO_2_ in EMEM supplemented with 10% FCS, 1% non-essential amino acids, 100 U/mL penicillin, 100 mg/mL streptomycin, and 2 mM L-glutamine. A total of 100,000 cells were seeded into each well of a 96-well plate and allowed to adhere for 1 h before the TCID_50_ assay was performed to determine viral replication.

### Virus

Throughout the experiments, the HSV-1 strain KOS (79) (kindly provided by the Section for Experimental Virology, Institute of Medical Microbiology, Jena University Hospital) was used. Virus propagation and titration experiments were carried out with GMK cell cultures. Fluorescence microscopy experiments were performed using the recombinant variant HSV-1/E70K EGFP (Institute of Infection Medicine, Christian-Albrecht University Kiel and University Medical Center Schleswig-Holstein, Kiel, Germany), which is based on the HSV-1 strain 17 containing the experimentally generated mutation E70K in the TK protein sequence expressing the reporter protein EGFP (64).

### Virus infection and titration

Confluent endothelial cells on 24-well plates were infected with the HSV-1 KOS strain or the HSV-1/E70K EGFP strain for 1 h in serum-free conditions with an m.o.i. of 5 or 10, respectively. Non-infected control cells were included in all experiments. After 1 h p.i., the virus-containing medium was replaced by fresh full growth medium, and cells were incubated for the indicated times.

To obtain samples for virus titrations, the plates containing infected endothelial cells and medium were stored at −20°C for 1 d and thereafter thawed at room temperature. The freeze-thaw cycle caused the cells to burst and release intracellular viruses into the medium. Samples containing intra- and extracellular viruses were briefly centrifuged at 20,000 × *g*. The supernatants were serially diluted and transferred to GMK reporter cells to determine the respective amounts of virus using the TCID_50_ assay. Here, the positive cytopathic effects correlating with the amount of virus are documented and used for calculating the virus titer according to the Reed and Muench’s method (80). Each experimental condition was tested in triplicates.

### Cell stimulation

To study the role of AMPK in HSV-1 infection, the AMPK activator MK-8722 was used. In addition, an inhibitor of the mTOR pathway, Torin-2, and an inhibitor of the Akt pathway, Akt inhibitor VIII, were employed. The respective compounds were dissolved and diluted in DMSO and for each condition an equal amount of solvent was added to non-treated cells. To study the AMPK-activating properties of MK-8722 under the conditions of infection experiments, time- and dose-dependent incubations of cells with MK-8722 were carried out in full growth medium. To check the effects on virus replication, endothelial cells were pretreated with MK-8722 for 1 h in growth medium before infections with HSV-1 were carried out. 1 h p.i., growth medium was renewed, and MK-8722 was re-added for further incubation up to 24 h. To study the effect of MK-8722 p.i., the compound was added 12 h p.i. for 12 h. To study lipid synthesis, pretreatment of cells with MK-8722 was performed for 24 h.

### Cell viability using Cell Counting Kit-8

Cell viability was analyzed via Cell Counting Kit-8 (CCK8; Sigma-Aldrich, #96992) assay. Endothelial cells were plated in a 96-well plate at a density of 10,000 cells per well and cultured overnight. The cells were pre-treated dose-dependently with MK-8722 (0.1, 1, and 10 µM), Akt VIII (5, 20, and 50 µM), and Torin-2 (50, 100, and 250 nM) along with the solvent controls for 1 h. After 1 h, the cells were infected with HSV-1 along with non-infected cells. Following 1 h of infection the medium was changed to fresh growth medium along with the compounds. 100 µL of CCK-8 solution was added to each well of the plate after 24  h p.i. and the plate was incubated at 37°C for 2  h. The absorbance of each well was measured at 450  nm using a microplate reader (FLUOstar Omega, BMG Labtech, Ortenberg). Cell viability was normalized to non-infected, non-treated cells.

### Western blot analysis

Endothelial cells were lysed on ice by a 15 min incubation in ice-cold 50 mM Tris buffer (pH 7.4) containing 2 mM EDTA, 1 mM EGTA, 50 mM NaF, 10 mM Na_4_P_2_O_7_, 1 mM Na_3_VO_4_, 1 mM DTT, 1% Triton X-100, 0.1% SDS, 1 mM PMSF, and 10 µL/mL protease inhibitor cocktail. Lysates were centrifuged at 700 × *g* for 6 min and protein concentration was determined in aliquots using the DC Protein Assay Kit (Bio-Rad, Feldkirchen, Germany). The remaining supernatants were supplemented with Laemmli buffer, subjected to SDS-PAGE (30–50 µg lysate protein/lane) and transferred onto polyvinylidene fluoride (PVDF) membranes. The membranes were blocked for 1 h in Tris-buffered saline/Tween (TBST) [20 mM Tris (pH 7.6), 137 mM NaCl, 0.1% (vol/vol) Tween 20] containing 5% non-fat dried skimmed milk. Then, incubation with primary antibodies diluted in TBST containing 5% BSA was performed overnight at 4°C. The next day, membranes were incubated with the respective horseradish peroxidase-conjugated secondary antibodies for 1 h. Signal detection was performed using the enhanced chemiluminescence (ECL) reagent (GE Healthcare, Chicago, IL, USA) or Western Lightning Plus-ECL reagent (Perkin Elmer, Waltham, MA, USA). Quantification of protein bands was carried out by densitometry using ImageJ software (NIH, Bethesda, MD, USA). For expression studies, ratios between proteins of interest and β-actin were calculated. Signals of phosphorylated proteins were normalized to the respective total protein signals.

### siRNA treatment

Endothelial cells were seeded in 24-well plates or 30 mm dishes at 50–70% confluency 1 d prior to transfection. Transfection was performed with 0.5 µg/mL non-targeting or specific siRNA using the SAINT-sRNA transfection Kit (Synvolux Therapeutics B.V. Leiden, The Netherlands). For each 30 mm dish, siRNA was diluted in 100 µL PBS, combined with 20 µL Saint-sRNA diluted in 80 µL PBS and finally supplemented with 800 µL M199 containing 0.25% HSA. Cells were washed twice with pre-warmed Hanks’ balanced salt solution (HBSS) before the transfection solution was added. After 4 h, 2 mL of growth medium was added, and the cells were cultured for 72 h before performing experiments. For 24-well plates, one-fifth of the volumes of transfection solution and medium was added per well. Downregulation efficiency for the targeted proteins was verified by western blotting.

### RNA isolation and reverse transcription for ICP4 and gB expression

Endothelial cells were pretreated with 1 µM MK-8722 or solvent control for 1 h and infected with HSV-1 for up to 24 h. Total RNA was isolated from cells after respective times p.i. Cells were lysed and RNA was isolated using the column-based method by the NucleoSpin RNA Kit, Macherey-Nagel (#740955.250) according to the manufacturer’s protocol. Equal RNA concentrations from each sample were converted to cDNA by RevT-PCR using the First Strand cDNA Synthesis Kit by Thermo Fischer Scientific (#K1612) according to the manufacturer’s protocol.

### DNA isolation for HSV-1

Endothelial cells were pretreated with 1 µM MK-8722 or solvent control for 1 h and infected with HSV-1 for up to 12 h. Cells were lysed and nuclear pellet was obtained after the respective times p.i. Nuclear DNA was isolated using Qiagen Genomic DNA, Blood and Cell Culture Kit (#13323) according to the manufacturer’s protocol. After binding of the genomic DNA to the Qiagen Genomic-tip containing resin using low-salt and appropriate pH conditions, DNA was eluted in a high salt buffer and concentrated by isopropanol precipitation. Equal amount of DNA from each sample was then used for qPCR analysis of HSV-1.

### Quantitative real-time PCR

The cDNA/DNA samples were used for real-time PCR (RT-qPCR) using the SYBR Green qPCR Master mix by Thermo Fischer Scientific (#K0222). The master mix consists of buffer, thermostable DNA polymerase, deoxyribonucleotide triphosphates (dNTPs), and the SYBR Green dye. Each sample was measured in triplicates for each specific primer in a 96-well plate. The reaction mixture consisted of 10 µL of SYBR Green master mix, cDNA and water and 2 µL of the specific primer pair in each well. PCR was performed for 30 cycles with 15 s at 95°C and 1 min at 60°C. The mean value of the triplicates per sample was normalized to the expression of the housekeeping gene (β-actin for cDNA and 18S rRNA for DNA) and represented as graphs.

### Fluorescence microscopy

Endothelial cells were pretreated with 1 µM MK-8722 for 1 h or left untreated and infected with HSV-1/E70K EGFP for 24 h p.i. Then, cells were washed with warm (37°C) PBS, fixed with 4% paraformaldehyde for 15  min, washed twice with PBS and permeabilized in PBS containing 0.1% Triton X-100 for 5  min. After two washing steps with PBS, Hoechst 33342 (1 µg/mL in PBS) was added for 10 min and cells were washed three times with PBS and once with H_2_O before mounting the coverslips on microscopic slides using Fluoromount-G. A LEICA DMi8 TCS SP8 inverted laser-scanning microscope operated with the Leica Application Suite X was used to acquire immunofluorescence images (Leica Biosystems, Wetzlar, Germany). Images in 512 × 512 pixel format were acquired using a HC PL APO CS2 63×/1.40 oil objective with GFP laser excitation at 488 nm and scan speed of 400 Hz. Image acquisition was single-blinded, randomized, and areas of cells were scanned by primarily using the brightfield illumination to avoid bias and unintentional photobleaching. Fluorescence intensity was normalized to non-treated infected cells.

### Flow cytometry

Endothelial cells were pretreated with 1 µM MK-8722 for 1 h or solvent control and infected with HSV-1/E70K EGFP for up to 24 h. After respective times p.i., HUVEC were washed with PBS, detached from the plate using trypsin solution and centrifuged at 1,000 rpm for 10 min. The supernatant was discarded and the cell pellet was resuspended in 1 mL of 0.1% paraformaldehyde solution and incubated for 20 min at 4°C. Cells were washed once with PBS, resuspended in 1 mL BD Stain Buffer solution, and analyzed via flow cytometry (BD FACSLyric).

### Acetate incorporation experiments

To investigate the effect of MK-8722 on lipid synthesis, the incorporation of [1–14C]-acetate into cells was measured. Cells in 12-well plates were treated with MK-8722 for 24 h. Four hours prior to the end of the stipulated time, growth medium was replaced by M199 supplemented with 0.25% free fatty acid-free BSA, 500 pM biotin, 50 µM carnitine, 680 µM L-glutamine, 2.5 µg/mL ECGS, and 0.5 µCi/mL [1–14C]-acetate. At the end of the incubation time, cells were washed thrice with Hepes buffer [10 mM Hepes (pH 7.4), 145 mM NaCl, 5 mM KCl, 1 mM MgSO_4_, 1.5 mM CaCl_2_, 10 mM glucose], scraped in 200 µL 50 mM Tris buffer, transferred to Eppendorf tubes, and vortexed. Cellular lipids were extracted by adding 500 µL methanol and 250 µL chloroform. After 15 min incubation, phase separation was achieved by applying 250 µL chloroform, 250 µL 0.1 M KCl, and 2 µL citric acid. Samples were vortexed for 1 min, incubated for 5 min on ice, and then centrifuged at 500 × *g* for 5 min. The lower chloroform phase was taken out with a syringe and transferred to scintillation vials. Chloroform was evaporated and 0.5 mL 1% Triton X-100 was added to solubilize lipids. The vials were vortexed and left at room temperature for 1 h before 5 mL of scintillation cocktail was added. After mixing the vials, radioactivity was measured by a scintillation counter and the incorporation of [1–14C]-acetate was determined. Parallel samples were lysed using solubilization buffer (0.4% NaOH, 2% Na_2_CO_3_, 1% SDS) and protein content was determined using the DC Protein Assay Kit (Bio-Rad, Feldkirchen, Germany). Measured radioactivity of [1–14C]-acetate related to protein content was calculated and normalized to the untreated control.

### Statistical analysis

Information regarding number of experiments used for each experiment is given in the figure legends. Paired two-tailed Student’s *t*-test or one-way or two-way repeated measurements analysis of variance (ANOVA) were used for significance calculation after variance analysis with Holm–Šidák correction for multiple comparisons using Sigmaplot 14.5. *P* < 0.05 was considered as significant. Data in the figures are represented as mean + SEM (or mean ± SEM for line charts) of independent experiments performed with cells from different donors.

## SUPPLEMENTAL MATERIAL

Fig. S1 to S3 spectrum.00417-23-s0001.pdf Fig. S1: gB, ICP4 time dependence western blot. Fig. S2: cell viability assay. Fig. S3: EGFP FACS time dependence.

ASM does not own the copyrights to Supplemental Material that may be linked to, or accessed through, an article. The authors have granted ASM a non-exclusive, world-wide license to publish the Supplemental Material files. Please contact the corresponding author directly for reuse.
