# Supplementary material for: AMPK protects endothelial cells against HSV-1 replication via inhibition of mTORC1 and ACC1
Source: Microbiol Spectr. 2023 Sep 13;11(5):e00417-23. doi: 10.1128/spectrum.00417-23 (PMC10580915; doi:10.1128/spectrum.00417-23)
Supplement: Fig. S1 to S3 — Fig. S1: gB, ICP4 time dependence western blot. Fig. S2: cell viability assay. Fig. S3: EGFP FACS time dependence. [file spectrum.00417-23-s0001.pdf]

AMPK protects endothelial cells against HSV-1 replication via inhibition of mTORC1 and ACC1

Heena Doshi, Katrin Spengler, Amod Godbole, Yi Sing Gee, Jonathan Baell, Jonathan S Oakhill, Andreas Henke, Regine Heller

Correspondence to: regine.heller@med.uni-jena.de

Supplementary information

This file includes:

Supplementary Figures 1 to 3.

Supplementary Figures

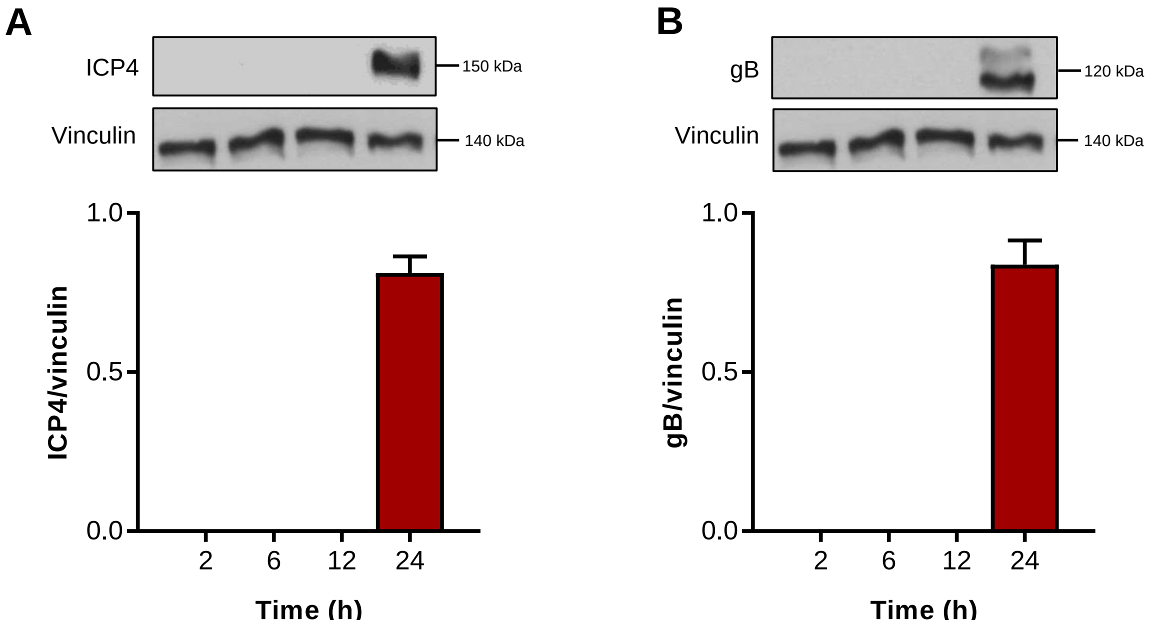

Fig S1: HSV-1-related proteins are detectable 24 h after infection in endothelial cells

HUVEC were infected with HSV-1 for the indicated times, lysed and subjected to western blot analyses. Representative immunoblots and densitometry analyses for ICP4 (infectious cell protein-4 (A)) and gB (glycoprotein B (B)) normalized to vinculin are shown, n=4.

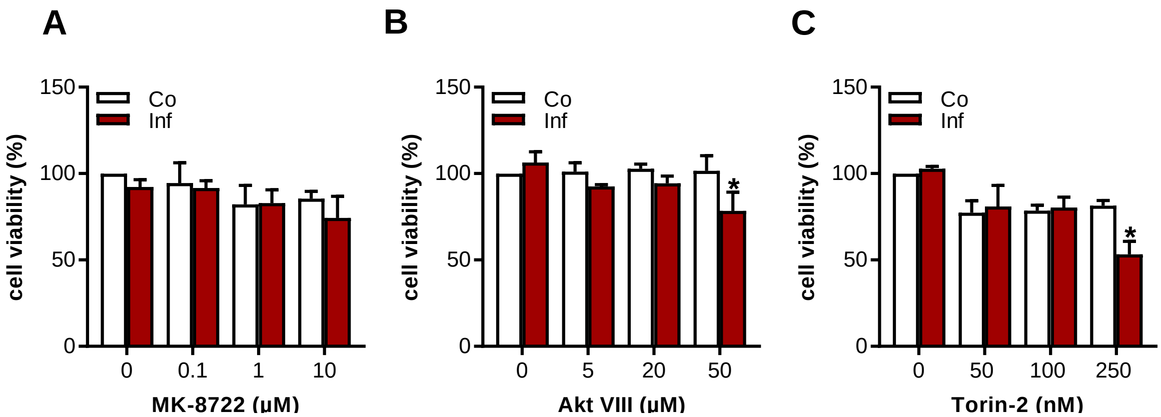

Fig S2: MK-8722, Akt VIII and Torin-2 do not affect endothelial cell viability at the applied doses

HUVEC were pretreated with the indicated concentrations of MK-8722 (A) Akt inhibitor VIII (B) or Torin-2 (C) for 1 h and infected with HSV-1 for 24 h (Inf) or left non-infected (Co). After 24 h cell viability was determined using the CCK-8 assay kit and normalized to values obtained in non-infected, non-treated cells (Co), for the respective experimental series, n=3. *$p<0.05$ vs. non-treated control using two-way repeated measurement ANOVA corrected via Holm–Šidák method.

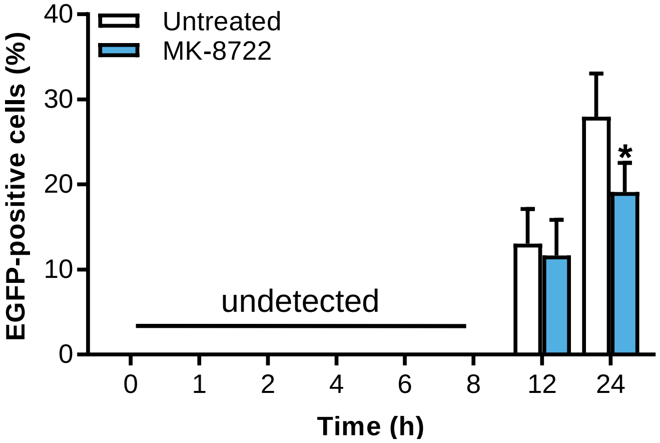

Fig S3: MK-8722 reduces the number of HSV-1-infected cells

HUVEC were pretreated with 1 µM MK-8722 for 1 h and infected with the HSV-1/E70K EGFP strain (10 m.o.i.) for the indicated times. The number of EGFP-positive cells was determined in flow cytometry, n=5. *p<0.05 vs. untreated infected cells using two-way repeated measurement ANOVA corrected via Holm–Šidák method.
